# Screening-based discovery of *Aspergillus fumigatus* plant-type chitinase inhibitors

**DOI:** 10.1016/j.febslet.2014.07.015

**Published:** 2014-08-25

**Authors:** Deborah E.A. Lockhart, Alexander Schuettelkopf, David E. Blair, Daan M.F. van Aalten

**Affiliations:** a Division of Molecular Microbiology, College of Life Sciences, University of Dundee, Dundee DD1 5EH, United Kingdom; b MRC Protein Phosphorylation and Ubiquitylation Unit, College of Life Sciences, University of Dundee, Dundee DD1 5EH, United Kingdom

**Keywords:** HTS, high-throughput screen/screening, GlcNAc, *N*-acetylglucosamine, *Af*ChiA1, *Aspergillus fumigatus* chitinase A1, *Sc*CTS1, *Saccharomyces cerevisiae* chitinase 1, *Af*ChiB, *Aspergillus fumigatus* chitinase B1, AMCase, acidic mammalian chitinase, *Hs*CHT, *Homo sapiens* chitotriosidase (chitinase 1), PI, percentage inhibition, Chitinases, Inhibitors, High-throughput screen (HTS), *Aspergillus fumigatus*

## Abstract

A limited therapeutic arsenal against increasing clinical disease due to *Aspergillus* spp. necessitates urgent characterisation of new antifungal targets. Here we describe the discovery of novel, low micromolar chemical inhibitors of *Aspergillus fumigatus* family 18 plant-type chitinase A1 (*Af*ChiA1) by high-throughput screening (HTS). Analysis of the binding mode by X-ray crystallography confirmed competitive inhibition and kinetic studies revealed two compounds with selectivity towards fungal plant-type chitinases. These inhibitors provide new chemical tools to probe the effects of chitinase inhibition on *A. fumigatus* growth and virulence, presenting attractive starting points for the development of further potent drug-like molecules.

## Highlights

• We performed a high-throughput screen of 60,000 compounds against *A. fumigatus* chitinase A1.
• Novel low micromolar competitive inhibitors were identified.
• These represent the most potent selective plant-type *A. fumigatus* chitinase inhibitors to date.
• We provide new tools for probing chitinase inhibition in *A. fumigatus* and other fungi.

## 1 Introduction

*Aspergillus fumigatus* is a filamentous opportunistic fungus and regarded as one of the most pernicious pathogens in immunosuppressed individuals. The spectrum and burden of clinical disease due to *A. fumigatus* is becoming increasingly recognised [1], [2]. Neutropenia in patients with haematological malignancies remains an important risk factor for invasive pulmonary aspergillosis (IPA). Despite therapeutic management, overall mortality remains around 50% for IPA [1], increasing up to 90% in disseminated disease [3]. Evidence is emerging that chronic pulmonary aspergillosis, affecting approximately 3–5 million patients globally [1], [4], may be alleviated by adjunct long-term oral antifungal therapy [5]. These contrasting infections rely on a limited repertoire of antifungal classes (polyenes, azoles and echinocandins), none of which are without significant drawbacks in terms of toxicity, drug–drug interactions and/or efficacy [6], [7], [8]. Azole resistance through one of several lanosterol 14 α-demethylase (*cyp*51A) mutations or environmental sources is an increasing concern [9]. With no new antifungal classes in the immediate pipeline, what is urgently needed is the characterisation of targets possessing novel modes of action [10].

The fungal cell wall is a complex polysaccharide composed predominantly of chitin, β-(1,3) glucan and galactomannan that provides structural stability and is essential for survival [11]. Chitin, a linear polymer of β-(1,4) linked *N*-acetylglucosamine (GlcNAc) forms the rigid inner layer of the cell wall and is also partially hydrolysed during morphogenesis [11]. This is performed by glycoside hydrolase family 18 (GH18) chitinases that catalyse the cleavage of β-(1,4) glycosidic bonds between GlcNAc residues. While GH18 chitinases are found in all kingdoms of life [12], chitin is absent in mammalian cells and these enzymes have been considered potential drug targets [13].

In *A. fumigatus* there are 17 chitinase genes phylogenetically divided into three subgroups forming two broad families [14], [15], [16]: subgroup B “plant-type” (*chiA*1–5, class III) and subgroup A/C “bacterial-type” (*chiB*1–12, class V). The latter, found most frequently in bacteria and also humans, are almost exclusively soluble secreted enzymes with exochitinase activity and unclear physiological function [11]. In contrast, “plant-type” enzymes are predominantly cell wall associated endochitinases. These endochitinases are potentially attractive antifungal targets, as they (i) provide selectivity since the distant human orthologues are non-essential and (ii) are extracellular. Nevertheless, chitinases pose a formidable challenge as a family containing presumably redundant genes. Comprehensive genetic validation involving targeted disruption of all 17 genes in a single strain is not feasible. Neither a quintuple mutant deleting all five plant-type chitinase genes [16] nor a single deletion of *chiB*1 in *A. fumigatus* yielded a significant phenotype under standard growth conditions [17]. Interestingly, recent work suggests chitinases may be involved in biofilm maturation [18]. A fungal biofilm is defined as a surface associated, highly structured community of hyphae encased by a polysaccharide extracellular matrix [19], [20]. Most clinical infections are biofilm related and, taken together, this finding sparked further investigation into chitinases as potential antifungal targets. A crucial first step is the generation of potent chemical tools to probe concurrent inhibition of all *A. fumigatus* chitinases, and thus allow for investigation of chemical validation as an alternative to genetic validation.

The natural product allosamidin was the first chitinase inhibitor reported [21]. This pseudotrisaccharide structurally mimics chitin and competitively inhibits all characterised GH18 family chitinases, albeit in the mid-micromolar range for the plant-type chitinase class. Limited availability and unfavourable chemical characteristics preclude use as a tool for chemical validation. Crucially, within the separate two families, *A. fumigatus* chitinases possess highly conserved active sites [22], [23], suggesting that it may be possible to generate separate pan-*Af*ChiA*x* and pan-*Af*ChiB*x* inhibitors that could be combined to investigate the phenotype of inhibiting all 17 chitinases concurrently. While inhibitors originally designed to inhibit *Af*ChiB1 also potently inhibit other “bacterial-type” chitinases [22], [24], a potent nanomolar *Af*ChiA*x* inhibitor to allow chemical validation of this attractive class of targets remains elusive. Natural product derivatives based on fragments of the bacterial-type chitinase inhibitor argifin yielded micromolar inhibitors of *A. fumigatus* chitinase A1 (*Af*ChiA1) [23]. Another study screened a model fungal plant-type chitinase, *Saccharomyces cerevisiae* CTS1 (*Sc*CTS1) against a library containing 880 off-patent drugs [25]. Despite extensive optimisation through structure-based activity relationships, derivatives failed to improve the potency of the parent compound, acetazolamide (*Af*ChiA1 IC_50_ 164 μM) [26].

Having exhausted all previous tractable chemical starting points we performed a high-throughput screen (HTS) of approximately 60,000 compounds against *Af*ChiA1. Here we describe the discovery of the most potent, novel, low micromolar scaffolds reported to date together with the crystal structures of the enzyme in complex with hits selective for plant-type chitinases.

## 2 Materials and methods

### 2.1 AfChiA1 cloning, expression and purification

*Af*ChiA1 (Arg28-His337) was expressed and purified as described previously [23]. In brief, the enzyme was expressed in *Pichia pastoris* as a secreted protein, the culture supernatant was dialysed, concentrated and *Af*ChiA1 purified using anion exchange followed by size exclusion chromatography. This protein sample was then used for the HTS, kinetics and structural biology described below.

### 2.2 AfChiA1 enzyme assay and high-throughput screen

Information according to published guidelines on the standardised reporting of HTS [27] is provided in Table S1. The Dundee Drug Discovery Unit (DDU) diversity set of 59,904 compounds was solubilised in DMSO (final maximum assay concentration of 1% (v/v) in all samples including controls) and a HTS performed in singlet at a concentration of 30 μM. Library compounds and DMSO controls were transferred to 384-well black polystyrene plates (Matrix) using a Hummingbird (Genomic Solutions). Columns 1–22 received library compounds, columns 23 and 24 were reserved for high/low controls (DMSO) and an 8-point standard inhibitory curve with acetazolamide (Sigma), a micromolar *Af*ChiA1 inhibitor [26]. Quality control (QC) plates, two per six assay plates, consisted of low control (columns 1–10), high control (columns 11–20) and a standard curve (columns 21–24). *Af*ChiA1 catalyses the hydrolysis of chitin and an existing assay liberating 4-methylumbelliferyl (4-MU) from the fluorogenic substrate 4-methylumbelliferyl β-d-*N*,*N*′,*N*″-triacetylchitotrioside (4-MU-GlcNAc_3_, Sigma) was optimised for microtitre plate format compatible with HTS [26]. *Af*ChiA1 activity was assayed in McIlvaine’s buffer (100 mM citric acid, 200 mM sodium phosphate [pH 5.5]) and 0.05 mg/ml BSA (Pierce) in a final reaction volume of 42 μl. Each reaction contained 10 nM *Af*ChiA1 except eight wells in column 24 (screening plates) and columns 1–10 (QC plates), to which only buffer was added to determine background signal. The reaction was initiated by 100 μM 4MU-GlcNAc_3_ and both additions were executed using a FlexDrop reagent dispenser (PerkinElmer). Assay plates were incubated on a microtitre plate shaker (Heidolph) at room temperature for 70 min. Fluorescence generated from the release of 4-MU was quantified using an EnVision 2102 multilabel reader (Perkin Elmer).

### 2.3 Data analysis

The Activity Base Suite (Abase) version 5.4 from IDBS was used for all data processing and analyses. For all compounds, the raw relative fluorescent units (RFUs) were corrected and normalised to percentage inhibition (PI) according to the equations in Table S1. Calculation of the *Z*-factor to determine the quality of the screening assay was as follows [28]:

$$Z\text{-factor}=1-\left[\left(3\;{\mathrm{SD}}_{\text{high control}}+3\;{\mathrm{SD}}_{\text{low control}})/({\mathrm{Mean}}_{\text{high control}}-{\mathrm{Mean}}_{\text{low control}}\right)\right]$$

for which SD_high control_ and SD_low control_ represent the standard deviations of the response from 8 to 12 control wells (uninhibited signal) and 8 to 12 background signal wells, respectively. Subsequent curve fitting to determine the IC_50_ was with XLFit version 4.2 (IDBS) using a four-parameter dose response curve.

### 2.4 Verification of primary hits and potency determination

Initial hits were (a) re-assayed at 30 μM for verification and (b) potency determined to generate an approximate IC_50_. Compounds were transferred into columns 1 and 13 of a 384-well polypropylene plate (Matrix) and serially diluted in 100% DMSO through 10 half-log increments in row orientation using a JANUS 8-channel Varispan automated workstation (PerkinElmer). This produced a source plate containing thirty test and two standard inhibitor curves (100× final assay concentration) in columns 1–10 and 13–22. An additional QC plate containing acetazolamide was also generated. From these source plates 0.5 μl, corresponding to a final concentration range of 30 μM to 1.5 nM, were transferred into replicate black 384-well polystyrene assay plates in duplicate. *Af*ChiA1 and substrate were added as in the original screen.

Confirmed hits were re-purchased from commercial sources (Table 1 shows the corresponding structure of each compound denoted in bold). Compounds **1**, **2**, **6** and **7** (ChemBridge Corporation, San Diego, CA, USA); compounds **3**, **4** and **8** (ChemDiv, San Diego, CA, USA); compounds **9** and **11** (Asinex Europe, Rijswijk, NED); compound **5** (Maybridge, Fisher Scientific, Loughborough, UK); compound **10** (InterBioScreen, Moscow, RUS) and compound **12** (Enamine, Monmouth, NJ, USA). For accurate duplicate potency determinations, the starting concentration was increased from 30 μM to 120 μM and diluted as described.

**Table 1:** Chemical structure of HTS compound hits identified against *Af*ChiA1 and corresponding inhibitory constants across other GH18 family 18 chitinases. Compounds were assigned to chemical series based on structural similarity.

Compound (series)	Structure	GH18 family chitinase IC50 (μM)				
		Plant-type		Bacterial-type		
		*Af*ChiA1^a^	*Sc*CTS1	*Af*ChiB1	AMCase	*Hs*CHT
**1** (1)		2.6	0.5	>100	9.8	>100
**2** (2)		34.9	N/D	N/D	N/D	N/D
**3** (3)		6.6	N/D	N/D	N/D	N/D
**4** (3)		19.4	>100	N/A	N/A	N/A
**5** (4)		9.2	2.4	>100	>100	>100
**6** (3)		11.4	63.0	>100	>100	>100
**7** (1)		14.3	>100	N/A	N/A	N/A
**8** (1)		16.2	>100	N/A	N/A	N/A
**9** (1)		11.8	>100	N/A	N/A	N/A
**10** (1)		45.3	>100	N/A	N/A	N/A
**11** (2)		58.8	>100	N/A	N/A	N/A
**12** (1)		23.7	16.1	65.5	13.6	>100

N/D, not determined. N/A, not applicable.

a The IC_50_ values quoted for *Af*ChiA1 are representative of the repeat potency determinations (performed in duplicate) and with the exception of compound **2** were within 5-fold of the original IC_50_.

### 2.5 Inhibition profiles across the GH18 family chitinases

Counter screening assays to determine an IC_50_ against other GH18 family chitinases included *S.* *cerevisiae* CTS1 (*Sc*CTS1, model plant-type chitinase) and three bacterial-type chitinases (*A. fumigatus* chitinase B1 [*Af*ChiB], acidic mammalian chitinase [AMCase] and *Homo* *sapiens* chitinase 1/chitotriosidase [*Hs*CHT]). Assays were performed as per *Af*ChiA1 with the following modifications [25], [29]. Bacterial-type chitinases (*Af*ChiB, AMCase and *Hs*CHT) utilised 4-methylumbelliferyl β-d-*N*-*N*-diacetylchitobioside (4MU-NAG_2_) as a substrate [29] and for these pentoxifylline (Sigma) was used as a control inhibitor [30]. Substrate concentrations for IC_50_ determinations were selected according to the published *K*_m_ values for each enzyme [25], [29], [31], [32]. The mode of inhibition was established by Lineweaver–Burk plots of steady-state kinetics with 50 μM to 1 mM of 4MU-GlcNAc_3_ in the presence of different concentrations of selected compounds [23].

### 2.6 Crystallography of AfChiA1 and ScCTS1 in complex with selected HTS hits

Native crystals were obtained using the hanging (*Af*ChiA1) and sitting drop (*Sc*CTS1) vapour diffusion methods using standard protocols [23], [25]. Crystals were soaked by the addition of 4 mM of the respective ligand (*Af*ChiA:compound **1** and *Sc*CTS1:compound **5**; prepared from a 0.2 M stock in 100% DMSO) to a crystallization drop. After incubation at 20 °C for 30 min, crystals were transferred to cryoprotectant (2.5 M Li_2_SO_4_ for *Af*ChiA1 and 0.1 M HEPES, 40% PEG 600 pH 7.5 for *Sc*CTS1) using a nylon loop for approximately 10 s before being flash frozen in liquid nitrogen. Diffraction data were collected at the European Synchrotron Radiation Facility (ESRF) in Grenoble, France. Diffraction data were processed and scaled with the HKL suite [33] to resolutions of 1.9 Å (*Af*ChiA:compound **1**) and 1.8 Å (*Sc*CTS1:compound **5**), respectively. Each complex was solved by manual molecular replacement with the apo structure of *Af*ChiA1 (PBD ID 2XVP [23]) or *S*cCTS1 (PDB ID 2UY2, [25]). Programs from the CCP4 suite [34] were used throughout the refinement process, which was initiated by rigid-body refinement and proceeded through iterative cycles of minimisation using REFMAC5 [35] and model building with Coot [36]. Ligand coordinates and topologies were produced with PRODRG [37]; ligands were not included until their conformations were completely defined by unbiased σA-weighted |*F*_o_| − |*F*_c_|, *φ*_calc_ electron density maps. Further refinement yielded the final models described in Table S2. Figures were generated using PyMol [38].

## 3 Results and discussion

### 3.1 High-throughput screening identifies novel AfChiA1 inhibitors

**Fig. 1:**
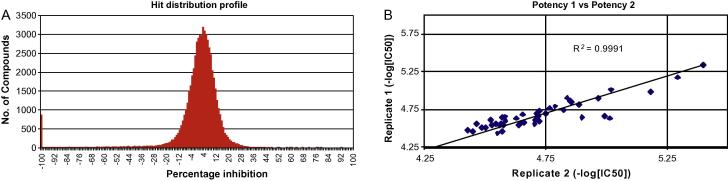
HTS of *Af*ChiA1 against the DDU diversity set. (A) Hit distribution profile representing percentage inhibition (PI) values for the HTS. Hits were designated as compounds that displayed a PI equal to or greater than two standard deviation units above the mean (35% inhibition). (B) Comparison of replicate potency determinations for the 48 primary screen hits. A *R*^2^ value of 1.0 indicates the regression line perfectly fits the data.

Further exploration of chitinases as potential antifungal targets requires new potent chemical tools for the plant-type subclass to complement the nanomolar inhibitor, bisdionin C, discovered for *Af*ChiB [39]. To identify new *Af*ChiA1 inhibitor scaffolds, a HTS was performed using a fluorescent assay. The Dundee Drug Discovery Unit (DDU) diversity set containing 59,904 compounds was screened against *Af*ChiA1 at 30 μM. This purpose-designed library adheres to lead-like properties of drug-like compounds [40]. The 172 assay screening plates generated a robust mean *Z* factor (±SD) of 0.79 (±0.05) indicative of an excellent assay with wide separation between the high and low controls [28]. The hit distribution profile (Fig. 1) showed nearly half of the library compounds (28,094/59,904) clustered around an *Af*ChiA1 inhibition of 1–10%. Primary screening identified 48 compounds with ⩾35% inhibition (0.08% hit rate) using greater than two times the standard deviation of the mean of the uninhibited control signal across all screening plates as a threshold. All 48 compounds were selected for re-confirmation and potency determination in duplicate (*R*^2^ = 0.9991, Fig. 1) using 10-point dose–response curves. From this, 23 compounds were verified hits with inhibition rates ⩾35% in two separate experiments giving an overall confirmed hit rate of 0.04%. Hits were ranked according to IC_50_ and 12 compounds produced values ⩽20 μM with the most potent hit (compound **1**, Table 1) returning an initial IC_50_ of 1.7 μM. Compounds were grouped into four chemical series according to their structural similarity (Table 1). Series 1 included the top hit and six additional compounds with a common heterocyclic core: a six-membered substructure fused to either a five- or six-membered ring. On the other hand, compound (**5**) was the sole member of series 4. After re-purchasing from the original vendor, hits were further evaluated for *Af*ChiA1 inhibition using a 10-point dilution series starting at 120 μM. The results were in accordance (<5-fold difference) with the original potency determinations with one exception (compound **2**, 15-fold increase).

### 3.2 Two compounds selectively inhibit plant-type chitinases in the low micromolar range

Of the confirmed hits identified by HTS, we wished to identify those selective for plant-type fungal chitinases in general. Given apparent redundancy in this enzyme class we postulated that scaffolds possessing pan plant-type chitinase inhibition provide more favourable tools for probing biological function than those with selectivity towards an individual enzyme within the family. The amino acid residues lining active site of the five plant-type chitinases in *A. fumigatus* are completely conserved apart from a single tyrosine residue (Y125) in *Af*ChiA1 [26]. Based on this, *Af*ChiA1 is considered a suitable model for the active sites of *Af*ChiA2–5. Additionally, we chose the well-characterised plant-type chitinase from *S. cerevisiae* (*Sc*CTS1), which has close homologs in *Candida albicans* (CHT1–3) [41] as well as, putatively, in *Aspergillus* *flavus*, *Aspergillus* *niger*, *Aspergillus* *clavatus* and *Aspergillus* *oryzae*, as a second ‘reference’ plant-type chitinase. To investigate our hypothesis, ten hits representative of each chemical series were evaluated for inhibition against two model plant-type chitinases. Of these, six compounds (**4**, **7**, **8**, **9**, **10** and **11**) did not show significant inhibition of *Sc*CTS1 with IC_50_ values exceeding 100 μM suggestive of discrepancies towards substrate binding and/or specificity between the two plant-type chitinases. Given our selection criteria these hits were not further evaluated. Previous work [25] revealed the active site pocket in *A. fumigatus* is shallower than in *S. cerevisiae* due to a large methionine (Met310) side chain and this may account for these hits having greater affinity for *Af*ChiA1 than *Sc*CTS1. On the other hand, four compounds (**1**, **5**, **6** and **12**) inhibited both *Af*ChiA1 and *Sc*CTS1 within half an order of magnitude suggestive of a common binding mode (Table 1) and pan plant-type chitinase activity. Compounds **1** and **5** are of particular interest with IC_50_ values <10 μM for both *Af*ChiA1 and *Sc*CTS1.

Next, we assessed the selectivity of compounds **1**, **5**, **6** and **12** across GH18 family chitinases. In particular we sought to identify compounds only with selectivity towards plant-type chitinases to complement existing nanomolar inhibitors of the bacterial-type chitinases. Counter screening was performed against three bacterial-type chitinases: *A. fumigatus* chitinase B1 [*Af*ChiB], acidic mammalian chitinase [AMCase] and *H. sapiens* chitinase 1/chitotriosidase [*Hs*CHT]). The most potent hit, compound **1**, had no activity against *Af*ChiB or *Hs*CHT, but inhibited AMCase with a fourfold drop-off in potency compared to *Af*ChiA1 (Table 1). Compounds **5** and **6** were selective for plant-type chitinases with IC_50_ values above 100 μM for all of the bacterial-type chitinases tested. Finally, compound **12** displayed comparable activity between both classes (Table 1) and was discarded from subsequent analysis. Although chitin is absent from humans, the human genome includes two active bacterial-type chitinases *Hs*CHT and AMCase. Despite extensive work the precise role of these enzymes is unresolved although they may offer a protective role [12]. Hence, we wished to focus on potent scaffolds that did not display selectivity towards these enzymes.

**Fig. 2:**
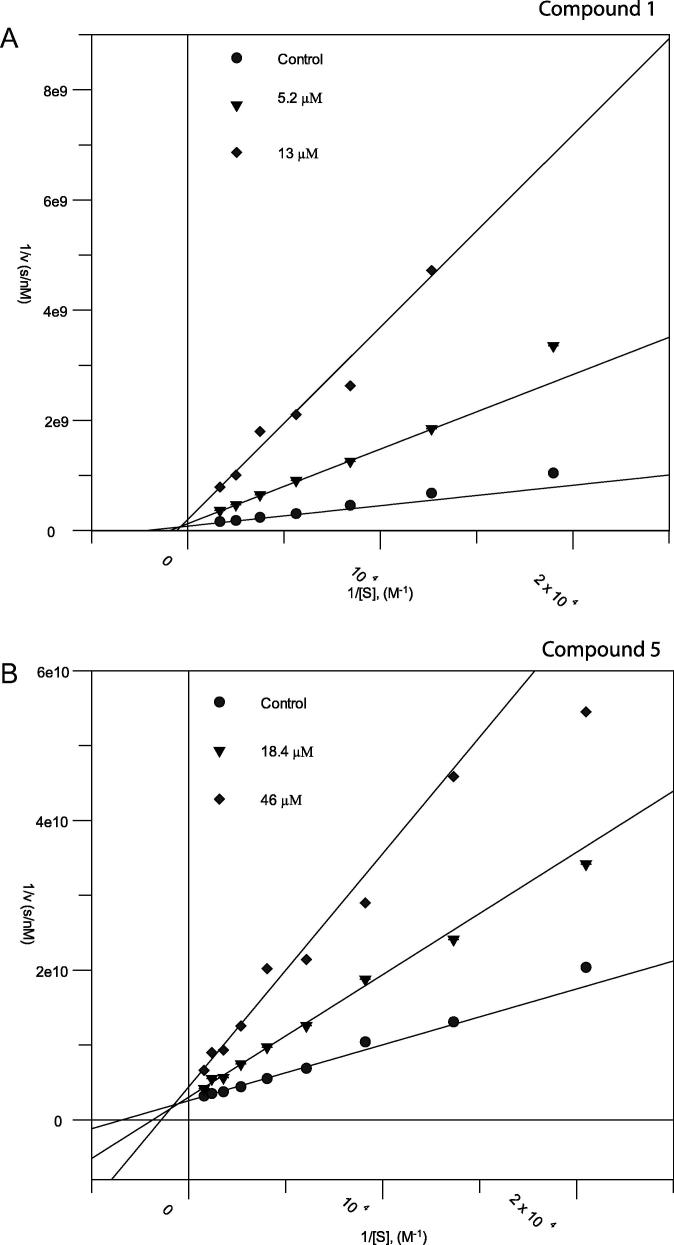
Lineweaver–Burk plots of compounds **1** (A) and **5** (B) measured against *Af*ChiA1 using different concentrations of each inhibitor. The data were fitted against a competitive inhibition model and resulted in a *K*_i_ of 1.2 μM (compound **1**) and 9.5 μM (compound **5**). *K*_m_ 300 ± 27 μM [23].

Additional kinetic experiments were performed on compounds **1**, **5** and **6** against *Af*ChiA1. Steady state kinetics were measured to determine the mode of inhibition and the corresponding inhibition constant, *K*_i_. Inhibitor concentrations were chosen according to the previously determined *Af*ChiA1 IC_50_ (Table 1). Lineweaver–Burk analysis indicates that each compound competes with the pseudo-substrate 4MU-GlcNAc_3_ for binding to the *Af*ChiA1 active site (Fig. 2). Inhibition constants were 1.2 μM, 9.5 μM and 10.6 μM for compounds **1**, **5** and **6** respectively.

### 3.3 Crystal structures of compound **1** and **5** reveal a competitive binding mode

**Fig. 3:**
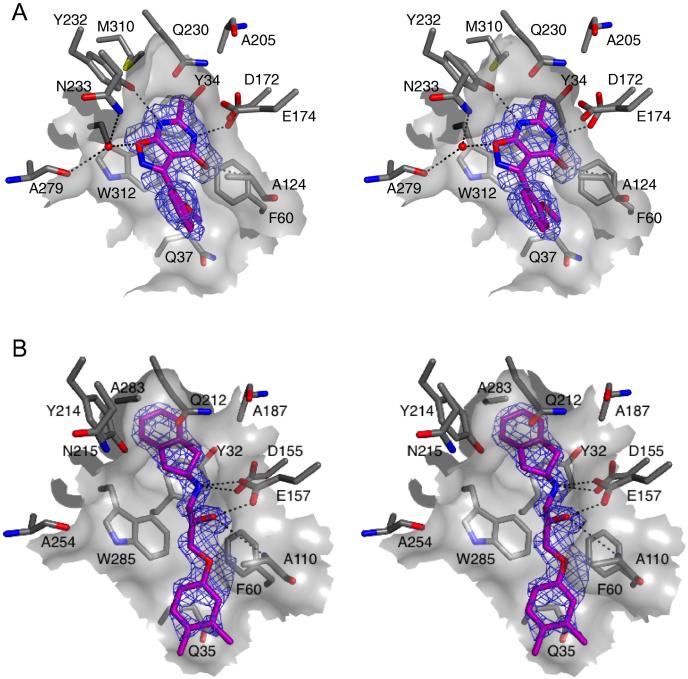
Stereo view of HTS hit compounds (purple) binding to the active site of plant-type chitinases. (A) Compound **1** bound to *Af*ChiA1. (B) Compound **5** bound to *Sc*CTS1. Surface representation of the protein active site is shown as a grey cartoon. Side chains of key amino acid residues interacting with the ligand (purple) are depicted as grey sticks and labelled. Potential hydrogen bonds are indicated by black dotted lines, a water molecule participating in indirect hydrogen bonding between compound 1 and AfChiA1 is represented as a red sphere. The unbiased (calculated before the addition of the ligand to the model) |*F*_o_| − |*F*_c_|, *φ*_calc_ electron density map covering each ligand (purple) is contoured at 3.0 σ.

As compounds **1** and **5** were thought to represent the most promising inhibitor scaffolds, we determined the molecular basis of binding to allow for the future design of derivatives. *Af*ChiA1 crystals were soaked with compound **1**, diffraction data were collected and scaled to 1.9 Å resolution, the *Af*ChiA1-compound **1** complex structure solved by molecular replacement and refined to an *R*_free_ of 0.22 (Table S2). Electron density corresponding to the ligand (compound **1**) was observed in the active site for both molecules in the asymmetric unit. As previously observed [26], the active site was partially obstructed in chain A by a symmetry-related protein molecule and further discussion focuses on chain B. Comparison of the *Af*ChiA-compound **1** complex with a published *Af*ChiA-acetazolamide complex (PBD 2XTK [26]) revealed similar overall conformations with an RMSD of 0.15 Å for 309 Cα atoms. Compound **1** consists of two ring systems, an isoxazolopyrimidine stacking on Trp312 and an attached methoxybenzene. The pyrimidine moiety inserts deeply into the *Af*ChiA active site and thus is likely most important for binding (Fig. 3A). The 6-methyl of the pyrimidine moiety inserts into a pocket at the bottom of the binding cleft that is formed by Tyr34, Met310, Ala205 and Gln230. Residues lining the *Af*ChiA1 active site form a number of hydrogen bonds with the ligand (Fig. 3A): the N1 ring atom donates a hydrogen bond to the side chain of Asp172, while the pyrimidine carbonyl group originating from the C2 position accepts a hydrogen bond from the backbone amine of Ala124 and the N5 ring atom accepts a third hydrogen bond from the side chain hydroxyl of Tyr232. The isoxazolo moiety points out of the binding cleft (Fig. 3A). It forms no direct hydrogen bonds with the protein, but engages in a (weak) water-mediated hydrogen bonding interaction with the side chain amide of Asn233 and the backbone carbonyl of Ala279. The methoxyphenyl moiety makes limited van der Waals contacts with the side chains of Trp312, Gln37, Phe60 and Ala124, but otherwise points towards the bulk solvent suggesting that the ring could be replaced. Crucially, compound **1** only interacts with side chains that are identical in all five *Af*ChiA enzymes. Strikingly the non-conserved Tyr125 side chain has flipped out to accommodate the methoxy group of the isoxazolo moiety and this may account for the improved inhibition of compound **1** over acetazolamide in *Af*ChiA1 by facilitating tight interactions of the neighbouring pyrimidine moiety within the depths of binding pocket. In *Sc*CTS1 a serine residue replaces the bulky flexible tyrosine unique to *Af*ChiA1 at the opening of the active site pocket. Interestingly both acetazolamide [26] and compound **1** displayed greater potency (8 and 5-fold respectively) against *Sc*CTS1 compared to *Af*ChiA1 providing contributory evidence that Tyr125 is not required for ligand specificity in plant-type chitinases.

Exhaustive attempts to obtain a complex of compound **5** with *Af*ChiA1 were unsuccessful. Due to comparable IC_50_ values between *Af*ChiA1 and *Sc*CTS1 (9.2 μM versus 2.4 μM, Table 1), as an alternative approach, we selected *Sc*CTS1 as a model to study this interaction. The beta-barrel containing the active site is highly conserved between *Sc*CTS1 (PDB 2UY2 [25]) and *Af*ChiA1 (PDB 2XVP [23]) with an RMSD of 1.2 Å for 270 Cα atoms providing further rationale for our choice of surrogate. Compound **5** was soaked into *Sc*CTS1 crystals and diffraction data were collected to 1.8 Å. The structure was solved by molecular replacement and refined to a final *R*_free_ of 0.23 (Table S2). Clear electron density for compound **5** was observed in the active site (Fig. 3B), corroborating the kinetics data showing that it, like compound **1**, is a competitive inhibitor. The *Sc*CTS1–compound **5** complex (Fig. 3B) shows the ligand filling a similar space to that occupied by compound **1** despite the lack of obvious chemical similarities. The dihydroindene moiety inserts into the same pocket as the pyrimidine methyl of compound **1**. The hydrogen bonding requirements of the Asp172 side chain (Asp155 in *Sc*CTS1) is satisfied by the linker amine of compound **5**, which also donates a potential hydrogen bond to the carboxylate of Glu157 (Glu174 in *Af*ChiA1). Continuing the ‘hydrogen bond zipper’, the linker hydroxyl of compound **5** also donates a hydrogen bond to the Glu157 side chain. Finally the ether oxygen of the ligand accepts a hydrogen bond from the Ala110 backbone (equivalent to Ala124 in *Af*ChiA1). An aromatic group stacking with the active site tryptophan side chain (Trp312 in *Af*ChiA1, Trp285 in *Sc*CTS1) is a feature common to both compound **1** and acetazolamide as well as numerous other family 18 chitinase inhibitors [26]. Consequently, the absence of stacking moieties in compound **5** is unusual, though it may help explain the specificity of this molecule for plant-type over bacterial-type family 18 chitinases, as the latter group generally possesses a second tryptophan that forms a ‘lid’ for the active site [39]. Bacterial-type chitinase inhibitors generally bind sandwiched between the two Trp side chains, suggesting that these enzymes may have a stronger preference for flat/aromatic ligands. Binding of the bulky dihydroindene deep in the *Sc*CTS1 active site induces a conformational change in the protein, with the side chain of Tyr214 (Tyr232 in *Af*ChiA1) and the following loop being pushed out by ∼2 Å compared to the superimposed *Sc*CTS1–acetazolamide complex structure. It is likely that binding of compound **5** to *Af*ChiA1 in an equivalent pose will require more dramatic conformational changes to the binding site, as the dihydroindene-binding pocket is smaller in *Af*ChiA1 due to the presence of Met310 (equivalent to Ala283 in *Sc*CTS1) It is possible that this explains the slight decrease in affinity of compound **5** for *Af*ChiA1 compared to *Sc*CTS1.

## 4 Concluding remarks

Chitinases represent a fascinating group of enzymes from a chemical perspective although their precise physiological roles remain elusive. In particular, the bacterial-type family have been extensively studied in terms of competitive inhibitors that mimic the carbohydrate substrate interaction [39], [42], [43]. Although both a natural product cyclopentapeptide inhibitor (argifin [43]) and a synthesised derivative based on linking a two-xanthine ring system (bisdionin C [39]) have high nanomolar affinities for *Af*ChiB they provide limited starting-points for plant-type chitinase inhibitors [23]. This is highlighted by fundamental differences in substrate specificity and active site architecture between the different families. Comparison of the crystal structures (*Af*ChiA1 PDB 2XVP [23] and *Af*ChiB1 PDB 1W9P [29]) of both chitinase subclasses in *A. fumigatus* reveals a deep pocket unique to plant-type chitinases in the base of the substrate binding groove. On the other hand, bacterial-type chitinases posses a more shallow and accessible groove with the tight binding of bisdionin C (*Af*ChiB IC_50_ 200 nM) attributed to tryptophan stacking beyond the single one conserved between *Af*ChiA1 and *Af*ChiB1 [39].

In contrast plant-type chitinases are underexplored in terms of chemical inhibitors. Prior to this study the only reported plant-type chitinase inhibitors were allosamidin [21], acetazolamide [26] and guanylurea derivatives [23], all of which are remarkably poor in terms of their inhibition against *Af*ChiA1. Structurally they are unfavorable: allosamidin extends beyond the conserved binding pocket while all attempts to obtain acetazolamide/guanylurea based derivatives failed to improve potency.

Our HTS against *Af*ChiA1 has provided novel, low micromolar plant-type chitinase competitive inhibitors (Table 1) that allow for tailored ligand specificity. Of the two hits for which we obtained structural information to illustrate the binding mode (Fig. 3), compound **1** (IC_50_ 2.6 μM), a novel pyrimidinone scaffold, could be a platform for chemical modification to inhibit a broad range of fungal plant-type chitinases and increase potency towards nanomolar inhibition. Specifically, as the 6-methyl moiety of the pyrimidine ring engages the depth of the *Af*ChiA1 binding pocket there may be scope for larger substituents in this position to fully occupy this space. This is supported by the known binding mode of acetazolamide, which interacts in the same pocket with an ethyl group. Taken together with information derived from the *Sc*CTS1-compound **5** complex, an ideal substitution could involve anything from a small methyl group to a more bulky ring in terms of size. Due to the tight affinity of the pyrimidine ring for the *Af*ChiA binding pocket and three key hydrogen bond interactions (Fig. 3A) no further extensions are possible. Currently the methoxyphenyl moiety provides little contribution to binding and therefore could be replaced. We hope these modifications will concurrently drive selectivity further towards the plant-type chitinases and create chemical tools to probe their biological role.

This work represents a significant advance in generating chemical starting points and provides a platform for the development of nanomolar *Af*ChiA1 inhibitors that are required to fully dissect the biological importance of these fungal plant-type chitinases. Evidence of an upregulation in plant-type chitinase activity in mature *A. fumigatus* biofilms is emerging suggestive of a role in the composition of the extracellular matrix potentially through the liberation of extracellular DNA [18]. Intriguingly acetazolamide, a weak plant-type chitinase inhibitor (*Af*ChiA1 IC_50_ > 150 μM) [25], [26], was shown to reduce *A. fumigatus* biofilm biomass [18]. If further work elaborating our novel pyrimidinone scaffold succeeds and the role of plant-type chitinases in *A. fumigatus* biofilm maturation is conclusive, this would open up translational prospects perhaps one day leading to the clinical use of chitinase-inhibitors as anti-biofilm agents.
