# Supplementary material for: Screening-based discovery of Aspergillus fumigatus plant-type chitinase inhibitors
Source: FEBS Lett. 2014 Aug 25;588(17):3282–90. doi: 10.1016/j.febslet.2014.07.015 (PMC4158421; doi:10.1016/j.febslet.2014.07.015)
Supplement: Supplementary data 1 [file mmc1.doc]

**Supplementary materials**

**Table S1**: *Af*ChiA1 high throughput screen protocol.

| **Category** | **Parameter** | **Description** |
| --- | --- | --- |
| **Assay** | Nature of assay | Fluorescence based assay that detects inhibition of an isolated molecular target. |
|  | Target | *Aspergillus fumigatus* chitinase A (*Af*ChiA1). |
|  | Primary measurement | Detection of 4MU fluorescence liberated from 4MU-GlcNAc_3_. |
|  | Key reagents (final concentration) | 1. McIlvaine’s buffer (100 mM citric acid, 200 mM sodium phosphate) pH 5.5 2. 0.05 mg/ml BSA (Pierce) 3. 10 nM *Af*ChiA (materials & methods) 4. 100 μM 4MU-GlcNAc_3_ (Sigma) |
|  | Assay protocol | Refer to materials & methods. |
| **Library** | Library size | 59,904 compounds. |
|  | Library composition | Purpose designed tractable starting points for medicinal chemistry programmes that maximize diversity, minimize over-representation of scaffolds and adhere to “lead-like properties”. |
|  | Source | University of Dundee Drug Discovery Unit (includes some commercially derived compounds). |
|  | Additional information on library design | Brenk et al. |
| **Screen** | Format | 384-well black polystyrene plates (Matrix). |
|  | Concentration tested (final) | 30 μM compound in 1% DMSO (Sigma). |
|  | Plate controls (all in 1% DMSO) | High control (uninhibited activity): key reagents only.  Inhibition control (8-point potency curve): key reagents and a standard inhibitor.  Low control (basal signal): key reagents *minus Af*ChiA. |
|  | Compound / reagent dispensing system | Compounds: Source plate - JANUS 8-channel Varispan automated workstation (PerkinElmer). Transfer to assay plate - Hummingbird 384-well 500 nl cassette (Genomic Solutions).  Reagents: FlexDrop (Perkin Elmer). |
|  | Detection instrument and software | Fluorescence detection: EnVision 2102 multilabel plate reader (excitation 340 nm and emission 460 nm).  Data processing and analysis: Activity Base version 5.4 (IDBS). |
|  | Assay validation/QC | Z-factor: 0.79 (*n* = 172).  Assay plates were created in batches of six and flanked by two quality control plates containing high, low and inhibition controls. |
|  | Correction factors | Removal of basal relative fluorescent units (Mean_high control_ – Mean_low control_) prior to normalization. |
|  | Normalization | Percentage inhibition (PI) = 100 – (100 x [corrected test sample RFU / average corrected high control*]) |
|  | Additional comments | * Average of 8-12 control values per assay plate. |
| **Post HTS analysis** | Hit criteria | Hits exceeding a defined selection cut-off threshold of inhibition (> 35%) were ranked according to PI. |
|  | Initial hit rate | 0.08% |
|  | Additional assay(s) | Compounds were retested by the original assay and potency determined (see materials and methods). |
|  | Confirmation of hit purity and structure  Additional information on hit purity | Compounds for subsequent investigation were re-purchased from the original vendor and assessed for purity (> 97%) and molecular mass by HPLC.  Brand et al. |

**Table S2**: X-ray crystallography data processing and refinement statistics of plant-type chitinase-compound complexes. Values in parenthesis correspond to the highest resolution shell.

|  | ***Af*ChiA1 + compound 1** | ***Sc*CTS1 + compound 5** |
| --- | --- | --- |
| **Resolution range (Å)** | 24.98 – 1.90 | 20.00 – 1.80 |
| **Space group** | I2_1_2_1_2_1_ | P2_1_2_1_2 |
| **Unit cell dimensions** | *a* = 76.40 Å,  *b* = 128.51 Å,  *c* = 212.10 Å | *a* = 72.77 Å,  *b* = 112.16 Å,  *c* = 37.16 Å |
| **No. of observed reflections** | 313520 (18694) | 131685 (2204) |
| **No. of unique reflections** | 81562 (5332) | 27572 (1164) |
| **Redundancy** | 3.8 (3.5) | 4.8 (1.9) |
| ***I* / σ(*I*)** | 16.1 (3.3) | 23.7 (5.6) |
| **Completeness (%)** | 99.3 (98.5) | 94.9 (61.5) |
| ***R*_merge_** | 0.056 (0.415) | 0.051 (0.152) |
| **No. of residues** |  |  |
| **Protein** | 619 | 288 |
| **Ligand** | 2 | 1 |
| **Solvent** | 793 | 210 |
| ***R*_work_, *R*_free_** | 0.15, 0.18 | 0.16, 0.19 |
| ***B*-factors (Å^2^)** |  |  |
| **Protein** | 18.1 | 18.9 |
| **Ligand** | 35.4 | 22.3 |
| **Solvent** | 30.6 | 26.0 |
| **r.m.s.d. from ideal geometry** |  |  |
| **Bond lengths (Å)** | 0.014 | 0.013 |
| **Bond angles (°)** | 1.58 | 1.49 |
